# Stress Granule Formation Attenuates RACK1-Mediated Apoptotic Cell Death Induced by Morusin

**DOI:** 10.3390/ijms21155360

**Published:** 2020-07-28

**Authors:** Ye-Jin Park, Dong Wook Choi, Sang Woo Cho, Jaeseok Han, Siyoung Yang, Cheol Yong Choi

**Affiliations:** 1 Department of Biological Sciences, Sungkyunkwan University, Suwon 16419, Korea; color9151@gmail.com (Y.-J.P.); dongwookchoi85@gmail.com (D.W.C.); swcho0628@naver.com (S.W.C.); 2 Soonchunhyang Institute of Medi-bio Science (SIMS), Soonchunhyang University, Cheonan, Chungcheongnam-do 31151, Korea; hanjs015@sch.ac.kr; 3 Department of Biomedical Sciences, Ajou University Graduate School of Medicine, Suwon 16499, Korea; yangsy@ajou.ac.kr

**Keywords:** stress granule, morusin, PKR, eIF2α, RACK1, cell death

## Abstract

Stress granules are membraneless organelles composed of numerous components including ribonucleoproteins. The stress granules are characterized by a dynamic complex assembly in response to various environmental stressors, which has been implicated in the coordinated regulation of diverse biological pathways, to exert a protective role against stress-induced cell death. Here, we show that stress granule formation is induced by morusin, a novel phytochemical displaying antitumor capacity through barely known mechanisms. Morusin-mediated induction of stress granules requires activation of protein kinase R (PKR) and subsequent eIF2α phosphorylation. Notably, genetic inactivation of stress granule formation mediated by G3BP1 knockout sensitized cancer cells to morusin treatment. This protective function against morusin-mediated cell death can be attributed at least in part to the sequestration of receptors for activated C kinase-1 (RACK1) within the stress granules, which reduces caspase-3 activation. Collectively, our study provides biochemical evidence for the role of stress granules in suppressing the antitumor capacity of morusin, proposing that morusin treatment, together with pharmacological inhibition of stress granules, could be an efficient strategy for targeting cancer.

## 1. Introduction

Cells have evolved multiple strategies for maintaining cellular homeostasis in response to various extracellular stimuli. Among them are stress granules (SGs), unique subcellular compartments formed in response to environmental stressors, which are dynamically assembled and disassembled to determine cell fates via the coordinate regulation of many different biological processes [1,2,3].

SGs are non-membrane-bound cellular compartments, also called RNP granules due to a high enrichment of ribonuclear proteins [3]. This structure is dramatically induced under stress conditions including oxidative stress, heat shock response and viral infection where systemic shutdown of translation initiation occurs, implying a role for recapitulating various stress responses with anabolic processes [4,5,6]. The mechanisms underlying the microscopic formation of the SG structure have been well established [1,3]; nucleation during which the core structure is assembled by key components including G3BP1/2 and TIA1/Pub1, followed by pooling microstructures for growth to form mature biphasic SGs. During SG formation, recruitment and buildup of numerous substances including signaling molecules, metabolic enzymes, as well as untranslated RNA stalled in ribosomes has been observed [2]. Indeed, the SGs are a heterogenous structure, which is context- and stress-dependent [7]. For instance, G3BP1/2 is indispensable for SG formation in response to oxidative stress, while it is not upon osmotic stress [5,8]. Moreover, post-translational modifications of various proteins within SGs, including phosphorylation and arginine methylation, have been reported to contribute to the complexity of SG dynamics [9,10,11,12,13,14]. P-bodies are other RNP granules known to share many components with SGs, suggesting biochemical crosstalk between the two unique granule structures [15]. 

Many efforts have been made to understand the functional relevance of SG formation. Various biochemical evidence indicates cell-protective roles in different contexts: (1) SGs sequester various proteins including RACK1, TRAF2, and mTOR in order to attenuate the apoptotic cascade and lower hierarchy processes (e.g., specific anabolic pathways), thereby promoting efficient repair and consequent cell survival [16,17,18]. (2) Several proteins including PKR, RIG-1 and RNAseL are recruited to boost the innate immune response against viral infection [19,20].

Multiple lines of investigation indicating the SGs as a key determinant of cellular fate under various stress conditions have recently led to questions regarding the potential implication of SGs in human pathophysiology. In particular, interest in this topic has greatly increased following evidence that defects in SG assembly and clearance may be implicated in the pathogenesis of neurodegenerative diseases. For example, neuronal mTOR and autolysosome cascade play roles in SG assembly and disassembly, respectively, which may be relevant for the pathogenesis of Alzheimer’s disease [21]. SGs have also been implicated in the development, progression and metastasis of various types of cancers including lung carcinoma, pediatric medulloblastoma and sarcoma [22,23,24,25]. Therefore, in this context, development of efficient biochemical platforms for targeting SGs with an understanding of the relevant mechanisms remains an attractive area of research in this field. 

Morusin is a novel phytochemical isolated from the root bark of *Morus alba Linn* [26]. The beneficial effects of morusin have been described in many different contexts including inflammatory pulmonary diseases, diabetes, and neurocognitive diseases [27,28,29]. In particular, morusin has recently been highlighted for its antitumoral capacity in various human cancer cell lines [30,31,32,33,34,35,36]. The underlying mechanisms may involve inhibition of the STAT3/NF-κB pathway leading to caspase-3 activation and apoptotic cell death [37,38,39,40]. More recently, autophagy was revealed as a potential inhibitor of cell death induced by morusin [41]. Nevertheless, the full spectrum of biological pathways and cancer cell processes that either promote or restrain morusin-mediated cell death requires further investigation.

In this study, we provided biochemical evidence of SG induction by morusin, which in turn reduces morusin-mediated apoptotic cell death. Morusin-induced SGs appears to be PKR dependent, and result in RACK1 sequestration at the SGs, leading to protection against morusin-induced cell death. Altogether, this study proposes the cotreatment of morusin and SG inhibitor as a potential effective strategy for cancer treatment. 

## 2. Results

### 2.1. Morusin Induces Stress Granule Formation

We recently showed that autophagy inhibits morusin-induced cell death [41]. However, the survival mechanisms exploited by cancer cells in order to escape from cell death mediated by morusin are still largely unknown. This, together with the observation that SG formation reduces the cytotoxic capacity of several therapeutic reagents, led us hypothesize that cancer cells may induce SG formation in response to morusin treatment. 

**Figure 1:**
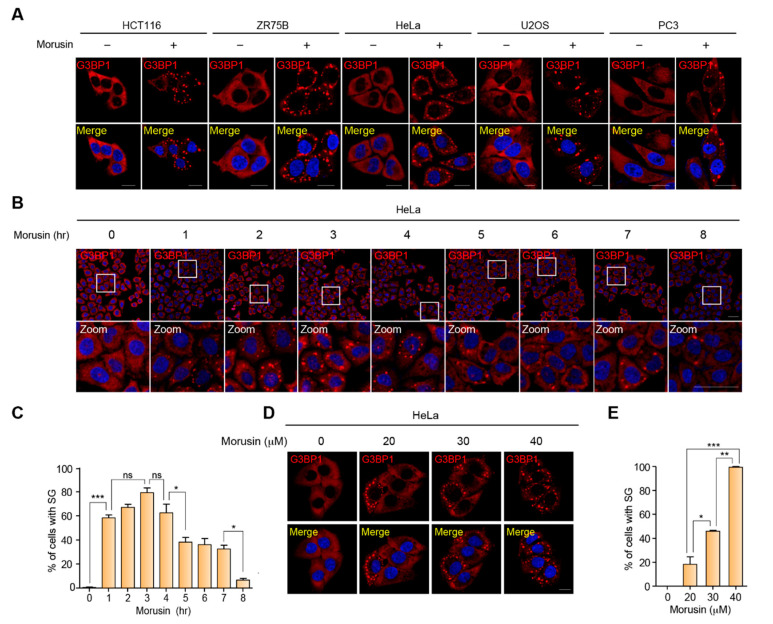
Morusin induces stress granules. (**A**) A representative image of immunostaining of G3BP1 in a panel of cancer cells treated with DMSO or 30 μM morusin for 3 h. Scale bar = 20 μm. (**B**) A representative image of immunostaining of G3BP1 in HeLa cells treated with DMSO or 30 μM morusin for 1 to 8 h at 1-h time intervals. Zoom indicates magnified images of white rectangles in the first row of images. Scale bar = 50 μm. (**C**) The graph displays the percentage of cells with G3BP1 puncta as in (**B**). (**D**) A representative image of immunostaining of G3BP1 in HeLa cells treated with DMSO or different concentrations of morusin (20, 30, and 40 μM) for 1 h. Scale bar = 20 μm. (**E**) The graph displays the percentage of cells with G3BP1 puncta as in (**D**). Data (**C** and **E**) are represented as the mean ± SEM and analyzed by one-way analysis of variance (ANOVA) followed by Bonferroni’s multiple comparison test (* *p* < 0.05, ** *p* < 0.01, *** *p* < 0.001 compared to the indicated points; *n* = 3).

To address this hypothesis, we first examined SG formation in morusin-treated cancer cells by immunostaining for G3BP1, a representative marker for SGs. Notably, dramatic induction of SGs was observed in the cells upon morusin treatment. The morusin-mediated SG induction was observed across all the cell lines tested, suggesting that this observation is not cell type specific (Figure 1A). In addition, we measured the temporal dynamics of SG formation at multiple time points of morusin treatment, showing that morusin-mediated induction of SGs occurred in 1 h and began to decrease 4 h after treatment (Figure 1B,C). Dose dependency was also tested at various concentrations used in previous studies (Figure 1D,E). Taken together, SG formation is acutely induced by morusin treatment at the range of concentrations where morusin exerts cytotoxicity in various cancer cell lines.

### 2.2. PKR Activation Is Required for the Induction of Stress Granules by Morusin

**Figure 2:**
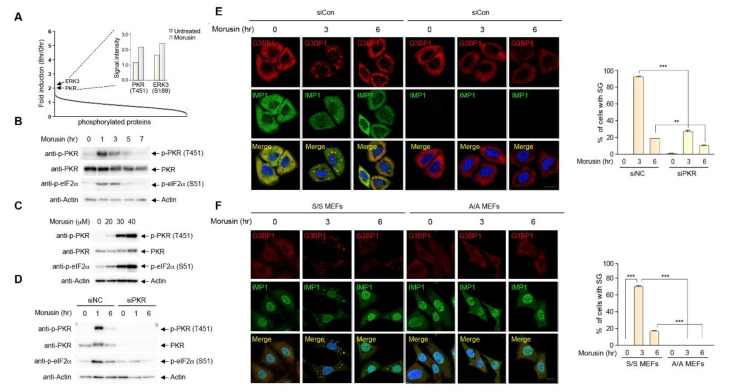
Protein kinase R (PKR) activation is required for stress granule induction by morusin. (**A**) Fold induction of protein phosphorylation upon morusin treatment measured by signal intensities of phospho-antibody arrays in HeLa cells treated with 30 μM morusin for 8 h normalized to those of DMSO-treated cells. Inset indicates relative signal intensities of PKR(T451) and ERK3(S189) phosphorylation. (**B**) A representative immunoblot analysis of PKR and eIF2α phosphorylation in HeLa cells treated with DMSO or 30 μM morusin for 1−7 h at 2-h intervals. (**C**) A representative immunoblot analysis of PKR and eIF2α phosphorylation in HeLa cells treated with DMSO or different concentrations of morusin (20, 30, and 40 μM). (**D**) A representative immunoblot analysis of PKR and eIF2α phosphorylation in HeLa cells transfected with control siRNA or PKR siRNA, and treated with DMSO or 30 μM morusin for 1 or 6 h. (**E**) A representative image of immunostaining of G3BP1 and PKR in HeLa cells transfected with control siRNA or PKR siRNA, and treated with DMSO or 30 μM morusin for 3 or 6 h. (**F**) A representative image of immunostaining of G3BP1 and IMP1 in wild type (*eif2α* S/S) or eIF2α Ser51Ala MEFs (*eif2α* A/A) treated with DMSO or 30 μM morusin for 3 or 6 h. The graph (**E**,**F**) displays the percentage of the cells with G3BP1 puncta. Data are represented as the mean ± SEM and analyzed by one-way analysis of variance (ANOVA) followed by Bonferroni’s multiple comparison test (** *p* < 0.01, *** *p* < 0.001 compared to the indicated points; *n* = 3). (**E**,**F**) Scale bars represent 20 µm.

To identify the biochemical mechanisms underlying morusin-induced SG formation, we employed an unbiased approach using a phospho-specific antibody array allowing for the identification of the phosphorylated protein(s) implicated in morusin-mediated SG formation (Figure S1A). Interestingly, activating phosphorylations of two protein kinases, PKR and ERK3, were induced upon morusin treatment (Figure 2A). ERK3 was a somewhat plausible target of morusin, given its association with STAT3/NF-κB signaling and apoptotic pathways which were previously associated with the antitumor effects of morusin [37]. However, ERK3 activation does not appear to be engaged in morusin-induced SG formation (Figure S1B). 

Thus, we specifically focused on PKR, as this kinase was reported to play a major role in viral response-mediated SG assembly by inducing eIF2α Ser51 phosphorylation [42], a key event for SG formation via increased ribosome stalling during translation. PKR phosphorylation was confirmed by immunoblotting at multiple time points and concentrations (Figure 2B,C). PKR phosphorylation was greatly induced 1 h after morusin treatment and by morusin concentrations up to 40 μM. These dynamics are very similar to the dynamics of SG formation upon morusin treatment shown in Figure 1. Consistent with the previous literature [43], eIF2α phosphorylation displays the same dynamics as PKR. PKR activation and subsequent eIF2α phosphorylation upon morusin treatment led us to examine whether PKR activation is causally relevant for SG formation. For this, eIF2α phosphorylation and SG formation were measured in cells depleted of PKR using siRNA targeting the *PKR* gene. We observed impaired eIF2α phosphorylation and SG formation following PKR depletion (Figure 2D,E), suggesting that PKR activation followed by eIF2α phosphorylation is a prerequisite for morusin-induced SG formation. This finding was further supported by using mouse embryonic fibroblasts (MEFs) isolated from mice with a homozygous mutation at the eIF2α phosphorylation site (Ser51Ala). Immunostaining of MEFs with two SG markers, G3BP1 and IMP1, indicated that morusin cannot induce SG formation in A/A *eIF2α* mutant MEFs where PKR-mediated eIF2α phosphorylation is blunted [44], while SG formation was observed in WT MEFs (S/S MEFs) upon morusin treatment (Figure 2F). These results suggest that morusin induces SGs in an eIF2α phosphorylation-dependent manner through PKR activation.

### 2.3. Morusin-Induced Stress Granules Enhance Cell Survival by Inhibition of Cell Death

**Figure 3:**
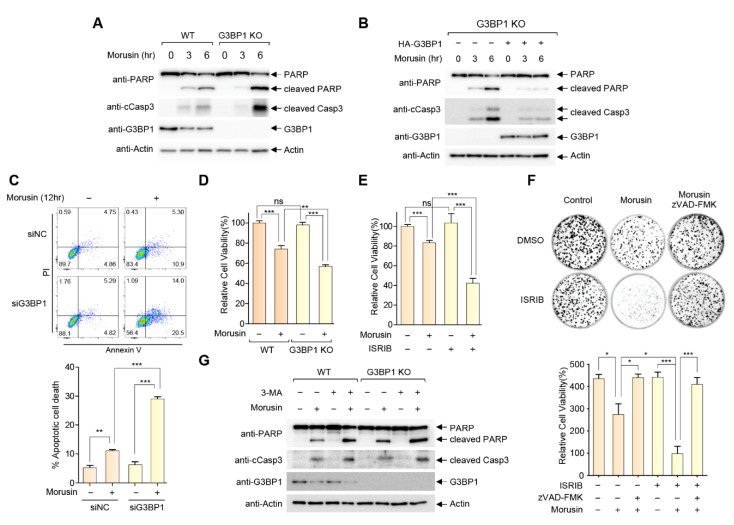
Morusin-induced stress granules enhance cell survival by inhibition of cell death. (**A**) A representative immunoblot analysis of PARP and caspase-3 cleavage in WT or G3BP1 KO HeLa cells treated with DMSO or 30 μM morusin for 3 or 6 h. (**B**) A representative immunoblot analysis of PARP and caspase-3 cleavage in G3BP1 KO HeLa cells transfected with empty vector or HA-G3BP1 treated with DMSO or 30 μM morusin for 3 or 6 h. (**C**) Analysis of morusin-induced apoptosis using flow cytometry. Wild-type or G3BP1-depleted HeLa cells were treated with 30 μM morusin for 12 h, and apoptotic cells were assessed by flow cytometry. Annexin V-positive apoptotic cells are presented on the graph. (**D**) Cell viability assays in WT or G3BP1 KO cells treated with 30 μM morusin for 9 h. (**E**) Cell viability assay in U2OS cells treated with 30 μM morusin in the presence or absence of 200 nM ISRIB for 6 h. (**F**) Colony forming assays in HeLa cells treated with 30 μM morusin in the presence or absence of 200 nM ISRIB and/or 20 μM zVAD-FMK for 12 h. The number of colonies were quantified using ImageJ software. (**G**) A representative immunoblot analysis of PARP and caspase-3 cleavage in WT or G3BP1 KO HeLa cells pretreated with DMSO or 3-MA for 1 h, and subjected to DMSO or 30 μM morusin treatment for 5 h. Data (**C**–**F**) are represented as the mean ± SEM. Data were analyzed by one-way analysis of variance (ANOVA) followed by Bonferroni’s multiple comparison test (* *p* < 0.05, ** *p* < 0.01, *** *p* < 0.001 compared to the indicated points; *n* = 3).

To determine whether SG induction affects the cytotoxic capacity of morusin, we generated and examined SG-defective HeLa cells lacking G3BP1 by CRISPR gene editing (Figure S2). Notably, G3BP1 KO cells display a higher level of PARP and caspase-3 cleavage upon morusin treatment compared to G3BP1 WT cells, suggesting that SG inhibition sensitizes the cancer cells to morusin (Figure 3A). The sensitivity of G3BP1 KO to morusin was recovered upon G3BP1 reconstitution, indicating that the sensitivity could be specifically attributed to the defects in G3BP1-mediated SG formation (Figure 3B). Morusin-induced apoptosis was determined by flow cytometry after annexin V-FITC and propidium iodide (PI) staining. Morusin treatment increased annexin V-positive cells, which were markedly potentiated in G3BP1-depleted cells (Figure 3C). Moreover, the observation was further supported by the cell viability assays in Figure 3D,E, where morusin-induced cell death was increased by 50% in G3BP1 KO cells and WT cells treated with integrated stress response inhibitor (ISRIB), a chemical inhibitor of eIF2α phosphorylation-mediated translational inhibition which thereby disrupted SG formation (Figure S3). We also found that the apoptotic pathway was significantly involved in SG-mediated inhibition of morusin, as the pan-Caspase inhibitor zVAD-FMK completely blocked morusin-induced cell death, both in WT and ISRIB-treated cells (Figure 3F). 

Autophagy regulation of SG clearance has been well established [45,46]. For example, autophagy inducing reagents such as rapamycin promote SG disassembly, while SG clearance was suppressed by wortmannin and 3-MA, chemical inhibitors of autophagy [47,48]. We employed 3-MA as a chemical tool to examine the effect of SG retention on morusin-mediated cell death. As shown in Figure 3G, 3-MA treatment facilitates morusin-induced PARP cleavage in WT cells, providing further support for the involvement of SGs in the cytotoxic effects of morusin. It should be mentioned that 3-MA treatment also synergizes with morusin for cell death even in G3BP1 KO cells, consistent with a previous study from our group showing the protective roles of autophagy in morusin-induced cell death [41].

### 2.4. RACK1 Sequestration at Stress Granules Inhibits Apoptosis

**Figure 4:**
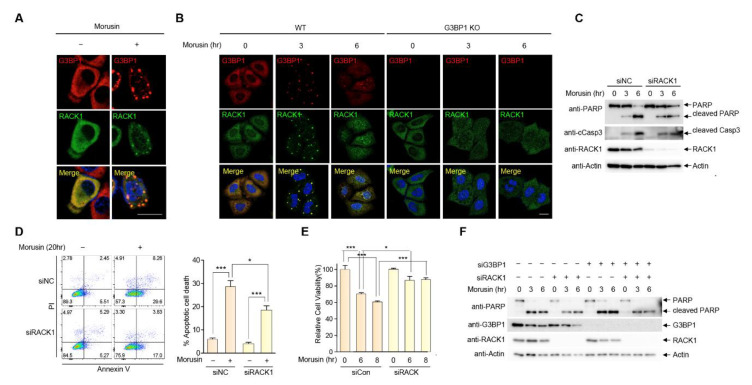
RACK1 recruitment into stress granules attenuates morusin-mediated apoptosis. (**A**) A representative image of immunostaining of G3BP1 and RACK1 in HeLa cells treated with DMSO or 30 μM morusin for 3 h. (**B**) A representative confocal image of immunostaining of G3BP1 and RACK1 in WT or G3BP1 KO HeLa cells treated with DMSO or 30 μM morusin for 3 or 6 h. (**C**) A representative immunoblot analysis of PARP and caspase-3 cleavage in U2OS cells transfected with control siRNA or RACK1 siRNA, and treated with DMSO or 30 μM morusin for 3 or 6 h. (**D**) Analysis of morusin-induced apoptosis using flow cytometry. Wild-type or RACK1-depleted U2OS cells were treated with 20 μM morusin for 20 h, and apoptotic cells were assessed by flow cytometry. Annexin V-positive apoptotic cells are presented on the graph. (**E**) Cell viability assay in U2OS cells transfected with control siRNA or RACK1 siRNA, and treated with DMSO or 30 μM morusin for 6 to 8 h. (**F**) A representative immunoblot analysis of PARP and caspase-3 cleavage in U2OS cells transfected with control siRNA or siRNAs targeting G3BP1 and/or RACK1 as indicated. Data (**D** and **E**) are represented as the mean ± SEM and were analyzed by one-way analysis of variance (ANOVA) followed by Bonferroni’s multiple comparison test (* *p* < 0.05, *** *p* < 0.001 compared to the indicated points; *n* = 3). (**A** and **B**) Scale bars represent 20 µm.

**Figure 5:**
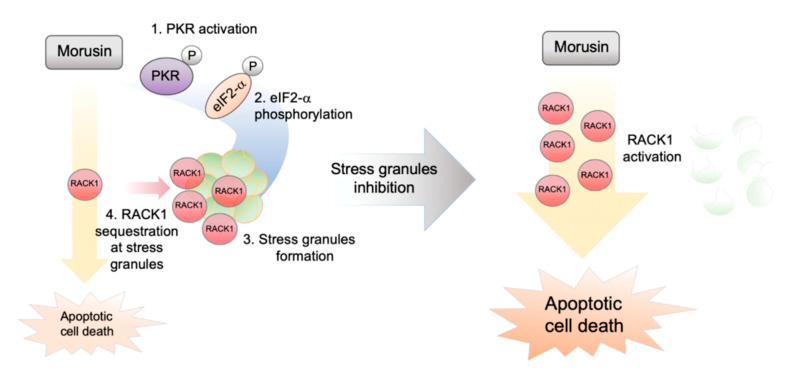
Proposed biochemical model for stress granule inhibition of apoptotic cell death induced by morusin.

It has been well established that SGs attenuate stress-induced cell death by trapping numerous proteins involved in intrinsic apoptotic pathways [4]. This mechanism involves the sequestration of receptors for activated kinase C-1 (RACK1), a proapoptotic protein that plays a role in the activation of the intrinsic apoptotic pathway [18]. This, together with the observation that activation of the intrinsic apoptotic pathway precedes cell death in morusin-treated cells, warranted further examination of the possibility that RACK1 may play a role in SG-mediated protection against morusin-induced cell death. Proteins recruited to SGs are greatly heterogenous according to the types of stressors, but we found that RACK1 is recruited to SGs in response to morusin, demonstrated by formation of a RACK1 speckle structure that colocalized with SGs in cells treated with morusin (Figure 4A). This result was further confirmed in morusin-treated G3BP1 KO cells where RACK1 displayed diffuse cytoplasmic localization upon morusin treatment (Figure 4B), suggesting that RACK1 is a component of SGs in response to morusin treatment. Next, we sought to address the functional relevance of RACK1 recruitment to SGs in this context. Surprisingly, morusin-mediated induction of PARP and caspase-3 cleavage is greatly impaired in RACK1-depleted cells using siRNA against RACK1 (Figure 4C). A similar result was observed when comparing apoptosis and cell viability between WT and RACK1-depleted cells (Figure 4D,E), suggesting that morusin-mediated cell death is significantly attributable to RACK1 activation. More importantly, sequestration of RACK1 appeared to be necessary for SG-mediated protection against the antitumor effects of morusin (Figure 4F). Immunoblotting indicated that increased PARP cleavage upon morusin treatment in G3BP1-depleted cells (lanes 8 and 9) was recovered in cells with depletion of both RACK1 and G3BP1 (lanes 11 and 12). Altogether, we suggest that morusin induces the formation of SGs where RACK1 is sequestered, leading to attenuation of the apoptotic capacity of morusin (Figure 5).

## 3. Discussion

Successfully established cancer cells are usually characterized by a remarkable capacity to adopt numerous biological processes that play roles in coping with multiple environmental stressors. Such functional flexibility is particularly beneficial for the survival of cancer cells with therapeutic intervention where activation of the relevant pathways attenuate cell death mediated by the therapy [49].

SGs have long been proposed as a key molecular platform for the coordinate regulation of various pathways to reduce cancer cell death mediated by a wide range of stressors and drugs. For example, SGs are induced in multiple cancer cell lines by treatment of chemotherapeutic reagents targeting RNA synthesis including 5-fluorouracil and sorafenib, thereby suppressing the antitumor capacity of the drugs [23,50]. However, questions about the drug-specific mechanisms of SG assembly have not yet been addressed. Indeed, the questions are important for targeted cancer therapies that induce SG formation, as components of SGs are highly heterogenous depending on the types of environmental stressors. Therefore, understanding the drug specific dynamics of SGs would be helpful for maximizing the efficiency and specificity of conventional interventions for cancers. In this context, our current study on the biochemical mechanisms underlying SG involvement in the antitumor capacity of morusin is noteworthy for several reasons. First of all, we successfully identified activation of the PKR-eIF2α axis as a molecular event critical for morusin-induced SG assembly. For this, we employed an unbiased approach using a phospho-specific antibody array. This is a relevant strategy since various post-translational modifications, and in particular, protein phosphorylation, have been thought to play key roles in SG formation and function. PKR has been implicated in SG formation upon activation of the innate immune response, which is critical for protection against viral infection. However, the understanding of PKR function in SG assembly in the context of cancer therapies has been very limited. This notion, together with our study showing the role of PKR in morusin-induced SGs, highlights the possibility of cotreatment of PKR inhibitor and morusin as an attractive strategy for targeting cancers. Second, we also found that morusin-mediated cell death requires an apoptotic cascade involving RACK1. RACK1 serves as a molecular scaffold for activation of several proapoptotic proteins including MKK7 and TRAF2, thereby promoting apoptotic cell death [51]. It was also suggested that RACK1 sequestration in SGs may be a key molecular event for SG-mediated inhibition of cell death [18]. However, the clinical implications of RACK1 sequestration within the SGs has been underappreciated. Therefore, any molecular intervention that specifically promotes liberation of RACK1 from the SGs may be relevant for boosting the antitumor capacity of therapeutic drugs that induce SG formation, including morusin.

Morusin is an isoprenylated flavone purified from the root bark of *Morus alba Linn*, exerting numerous health benefits including antinociceptive, antidiabetic, anticonvulsant, antibacterial, and antitumor activities [52,53,54]. In particular, the high antitumor capacity has been intensively tested across many different cancer cell lines. Here, we suggest that SG formation is a strategy for cancer cells to attenuate the cell death induced by morusin. Given that multiple cancer cells display different sensitivities of SG formation to various drugs and stressors, examining the capacity of SG formation of a targeted cancer would help determine an efficient strategy for morusin-mediated intervention for the particular cancer. 

Recently, our group showed that morusin increases autophagy, which in turn inhibits the antitumor capacity of morusin. We also proposed the underlying mechanism involving morusin-mediated regulation of the AMPK and mTOR pathway. Interestingly, multiple studies suggested that autophagy is a biological process indispensable for SG clearance, suggesting a potential link between SGs and autophagy in the context of morusin treatment. However, given the different temporal dynamics of SGs and autophagy (e.g., later time points of morusin treatment where SGs decrease and autophagy is activated), we speculated that the mechanisms underlying morusin involvement in the regulation of SG dynamics and its relevance regarding antitumor capacity may be more complicated than our current understanding. Therefore, how morusin recapitulates the multiple signaling pathways and its therapeutic relevance would be an attractive question to be addressed in the near future.

## 4. Materials and Methods

### 4.1. Cell Culture

HeLa, ZR75B, U2OS, and eIF2α Ser51Ala (*eif2α* A/A) and WT counterpart (*eif2α* S/S) MEFs were cultured in Dulbecco’s Modified Eagle’s Medium (DMEM) supplemented with 10% fetal bovine serum (FBS). HCT116 and PC3 cells were cultured in RPMI-1640 supplemented with 10% FBS. G3BP1 KO HeLa cells were cultured in DMEM supplemented with 10% FBS and 1 μg/mL puromycin. All cell lines were maintained in an incubator at 37 °C, 5% CO_2_.

### 4.2. Antibodies and Reagents

Antibodies and reagents used in this study were as follows: anti-G3BP1 antibody (BD Bioscience, 611126, Franklin Lakes, NJ, USA), anti-PKR (Santa cruz biotechnology, sc-6282, Dallas, TX, USA) anti-RACK1 (Santa Cruz biotechnology sc-17754), anti-phospho-eIF2α (Ser51) (Cell Signaling Technology, 9721S, Danvers, MA, USA), anti-cleaved caspase-3 (Cell Signaling Technology, 9661S), anti-PARP (Cell Signaling Technology, 9542S), anti-Actin (Cell Signaling Technology, 3700S), anti-PKR (Abcam, ab52506, Cambridge, MA, USA), anti-phospho-PKR(Thr451) (Abcam, ab81303), anti-IMP1 antibody (Bethel Laboratories, A303-424A, Montgomery, TX, USA), anti-HA-HRP (Roche, 12013819001, Mannheim, Germany), morusin (root bark of *Morus alba*) (Biopurity Phytochemicals Ltd., BP0961, Chengdu, Sichuan, China), ISRIB (Millipore sigma, SML0843, Saint Louis, MO, USA), 3-methyladenine (Millipore sigma, M9281), zVAD-FMK (Santa cruz biotechnology, sc-3067).

### 4.3. DNA and siRNA Transfection

Transfection of mammalian expression plasmids (HA-empty vector, HA-G3BP1, GFP empty vector and GFP-RACK1) into the indicated cells were performed using Lipofectamine 3000 Transfection Reagent (Invitrogen, L3000-015, Carlsbad, CA, USA), following the manufacturer’s instructions. Transfection of small interfering RNA (siRNA) into the indicated cells were carried out using Lipofectamine RNAiMAX reagent (Invitrogen, 13778-150) according to the manufacturer’s instructions. siRNA sequences used in this study were as follows: PKR siRNA, 5′-GACGG-AAAGACUUACGUUA-3′. ERK3 siRNA, 5′-GGCUUUUCAUGUAUCAGCU-3′, G3BP1 siRNA, 5′-CCAAGAUGAGGUCUUUGGUGGGUUU-3′. RACK1 siRNA, 5′-GGGAUGAGACCAACUAUGG-3′. Control siRNA, 5′-AACTGTCAGTCAGTCGTAGTA-3′.

### 4.4. Generation of G3BP1 KO Cell Line

The G3BP1 KO cell line was generated by CRISPR-Cas9-mediated genome editing. In brief, annealed oligonucleotides containing gRNA target sequence (5′-TAGTCCCCTGCTGGTCGGGC-3′) were cloned into the lenti-CRISPRv2 vector and transfected into HeLa cells. After 48 h of transfection, the cells were maintained on DMEM media supplemented with 2 μg/mL puromycin. Typical colonies were picked from the plates using cloning cylinders, subcultured, and expanded. Homozygous KO clones were characterized using genomic DNA sequencing, immunocytochemistry, and immunoblotting with anti-G3BP1 antibody.

### 4.5. Immunocytochemistry

Twenty-four hours after seeding cells onto coverslips in 6-well culture plates, the cells were treated with reagents as indicated in figures, followed by fixation of the cells using 3% paraformaldehyde. After 15 min incubation, cells were permeabilized using 0.1% Triton X-100 in PBS for 5 min, and were blocked with 1× phosphate-buffered saline containing 2% BSA for 20 min. Anti-G3BP1, anti-PKR, anti-IMP1, or anti-RACK1 antibody in 2% BSA were used for immunostaining of proteins in the cells. After 1 h incubation, samples were washed four times using PBS, and incubated with rhodamine-conjugated or Alexa Fluor 488-conjugated secondary antibody for 1 h at RT. The samples were washed four times using PBS and the nucleus was stained by DAPI (4′,6-diamidino-2-phenylindole). Confocal imaging was performed using a Zeiss LSM700 microscope and acquired images were processed with Adobe Photoshop.

### 4.6. Immunoblot Analysis

Cells were lysed in RIPA lysis buffer (20 mM Tris-HCl pH 7.5, 150 mM NaCl, 1 mM EDTA, 1% NP-40, 1% sodium deoxycholate, 5 mM *N*-ethylmaleimide, 1 mM Na_3_VO_4_). After 20 min incubation on ice, the lysates were pelleted at 14,000 rpm in a desktop centrifuge for 10 min at 4 °C. The supernatants were transferred to a new tube, and were prepared for immunoblotting using 5× sample buffer supplemented with 1% β-mercaptoethanol and subjected to SDS-PAGE (BioRAD system, 8–12% gels, Hercules, CA, USA). Proteins were transferred onto PVDF membrane and immunoblotted with the indicated antibodies, followed by detection with the indicated antibodies and ECL Western detection reagents (Intron, #16026, Seongnam-Si, Gyeonggi-do, Korea).

### 4.7. Cell Viability Analysis

Cell viability was assessed by the MTT (3-(4,5-dimethylthiazol-2-yl)-2,5-diphenyltetrazolium bromide) assay. Briefly, cells (1 × 10^3^ cells/well) were seeded onto 96-well plates and treated with morusin and/or ISRIB for 6–8 h. Viable cells were quantified by measuring the optical density at 595 nm using an enzyme-linked immunosorbent assay reader. The data are presented as the mean of biological quadruplicates ± SEM. 

### 4.8. Annexin V-FITC/Propidium Iodide Double Staining

Apoptotic cells were stained by using FITC Annexin V Apoptosis Detection Kit I (BD Biosciences, 556547). Cells were washed twice in 1× phosphate-buffered saline and incubated with 1× binding buffer (10 mM HEPES, 140 mM NaCl, 2.5 mM CaCl_2_, pH 7.4) containing 5 μL annexinV-FITC and 5 μL propidium iodide for 15 min at room temperature in the dark. The cells were washed with 1× binding buffer before analysis by flow cytometry. Data were collected on a BD Canto-II, FACSCalibur (BD Biosciences), and analyzed with FlowJo software (Tree Star, Ashland, OR, USA).

### 4.9. Colony Formation Assay 

For clonogenic survival assays, cells were seeded onto 60 mm plates (500 cells/plate). After 24 h, cells were treated with the indicated doses of morusin, ISRIB and zVAD-FMK, followed by visualization with Coomassie Brilliant Blue staining for counting the number of colonies.

### 4.10. Antibody Array

HeLa cells treated with DMSO or morusin were lysed in protein extraction buffer (Fullmoon biosystems, CA, USA), and the lysates were incubated with a prehydrated gel matrix column (Fullmoon biosystems). After 1 h incubation at RT, flow-through was collected into a tube, followed by protein quantification using the BCA protein assay kit (Pierce, Thermo Fisher Scientific, Waltham, MA, USA). For biotin labeling of proteins, 50 μg of lysates filled up to 75 μL with labeling buffer was incubated with 3 μL of biotin dissolved in DMF (10 μg/μL) for 90 min at RT with gentle agitation. The samples were treated with stop buffer (Fullmoon biosystems) for 30 min at RT to quench the reaction. For the antibody microarray, biotin-labeled samples in 6 mL of coupling solution were incubated with a preblocked antibody microarray slide (Fullmoon biosystems) for 2 h at RT with gentle shaking at 60 rpm, followed by 6 consecutive rounds of washing with 30 mL of washing buffer and one wash with Milli-Q grade water. The protein-bound array slide was treated with detection buffer supplemented with Cy3-streptavidin (0.5 μg/mL) (GE Healthcare). After 20 min incubation at RT with shaking, the slide was washed 6 times with 30 mL of washing buffer each for 5 min at RT with shaking, followed by a brief rinse with Milli-Q grade water. Slide scanning was performed using a GenePix 4100A scanner (Molecular Devices, LLC., San Jose, CA, USA). The slide was completely dried and scanned within 24−48 h. The scanned image was quantified with GenePix 7.0 Software.

## Supplementary Materials

Supplementary Materials can be found at https://www.mdpi.com/1422-0067/21/15/5360/s1.

## Abbreviations

SG	Stress granule
PKR	Protein kinase R
RACK1	Receptor for activated C kinase-1
CRISPR	Clustered Regularly Interspaced Short Palindromic Repeats
