# Supplementary material for: Stress Granule Formation Attenuates RACK1-Mediated Apoptotic Cell Death Induced by Morusin
Source: Int J Mol Sci. 2020 Jul 28;21(15):5360. doi: 10.3390/ijms21155360 (PMC7432505; doi:10.3390/ijms21155360)
Supplement: Supplementary file 1 [file ijms-21-05360-s001.pdf]

International Journal of
Molecular Sciences

MDPI

Supplementary

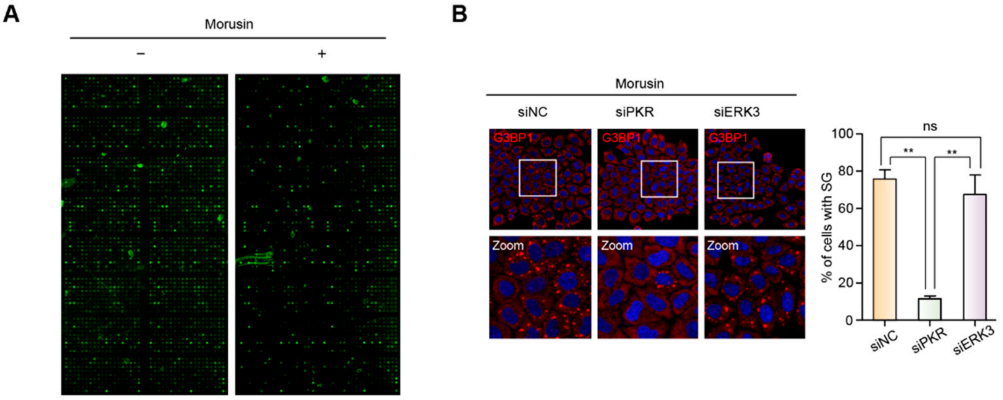

Figure S1. Phosphoprotein profiling of morusin-treated cells. (**A**) Raw data of phospho-specific antibody array in HeLa cells treated with DMSO or 30 μM morusin for 8 h. (**B**) A representative image of immunostaining of G3BP1 in HeLa cells transfected with siERK3 or siPKR, and treated with DMSO or 30 μM morusin for 3 h. Scale bar represents 50 μm.

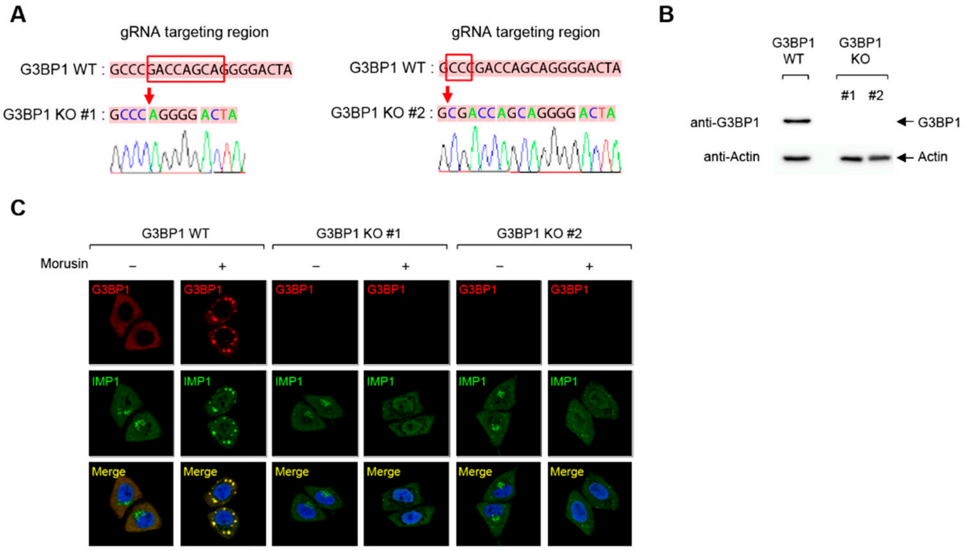

Figure S2. Stress granule formation is impaired in G3BP1 KO cell lines. (**A**) Validation of G3BP1 KO HeLa cell lines mediated by the CRISPR-cas9 system (#1 and #2) by sequencing the genomic region targeted by G3BP1 gRNAs. (**B**) Immunoblot image of G3BP1 in WT or G3BP1 KO HeLa cells. (**C**) A representative image of immunostaining of G3BP1 in WT or G3BP1 KO HeLa cells treated with DMSO or 30 μM morusin for 3 h. Scale bar represents 20 μm.

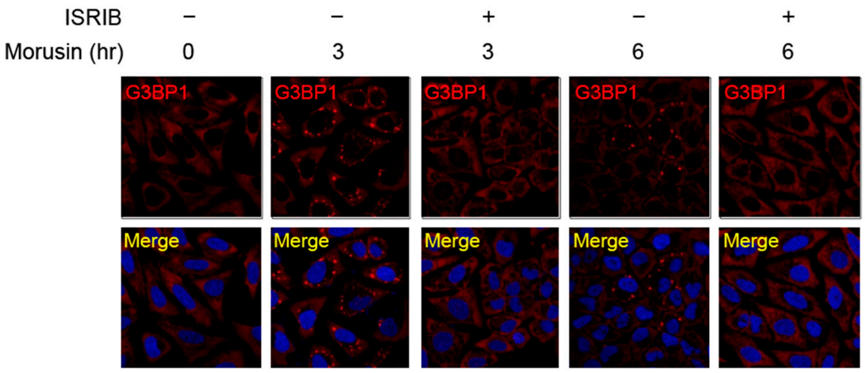

Figure S3. Morusin-induced stress granules require phosphorylated eIF2α-mediated translation repression. A representative image of immunostaining of G3BP1 in U2OS cells treated with DMSO or 30 μM morusin for 3 or 6 h, in the presence or absence of 200 nM ISRIB. Scale bar represents 50 μm.
